# Nerve-sparing resection of cervical vagal schwannoma with complete functional recovery: a case report

**DOI:** 10.1093/jscr/rjag916

**Published:** 2026-10-14

**Authors:** Zaid A Alzaghal, Faryal Imran, Mohammad Aamir Qayyum Sarguroh Khan, Aafreen A Nyaz, Gyanendra Jora, Safwan A Alzaghal, Hutham K Aliessa, Husam M Saleh

**Affiliations:** NMC Royal Hospital, Khalifa City, Abu Dhabi 35233, United Arab Emirates; NMC Royal Hospital, Khalifa City, Abu Dhabi 35233, United Arab Emirates; Sheikh Khalifa Medical City, Abu Dhabi 51900, United Arab Emirates; NMC Royal Hospital, Khalifa City, Abu Dhabi 35233, United Arab Emirates; KIST Medical College and Teaching Hospital, Lalitpur, Nepal; University of Sharjah, Sharjah, United Arab Emirates; Ajman University, Ajman, United Arab Emirates; NMC Royal Hospital, Khalifa City, Abu Dhabi 35233, United Arab Emirates

**Keywords:** vagal schwannoma, Schwann cells, peripheral nerve tumor, lateral neck mass

## Abstract

Vagal schwannomas are rare benign neoplasms arising from the Schwann cells of the vagus nerve, representing 2%–5% of all peripheral nerve tumors and ~10% of head and neck schwannomas. They typically present as slow-growing, asymptomatic lateral neck masses and pose significant diagnostic and surgical challenges due to their proximity to critical neurovascular structures. We report a case of a 28-year-old Asian male who presented with a right-sided neck swelling. Imaging workup including ultrasound, magnetic resonance imaging, and computed tomography angiography identified a well-defined mass in the right carotid space at the level of C4–C5, displacing adjacent vascular structures. The tumor was surgically excised en bloc via a lateral transverse approach with full preservation of vagal nerve function, confirmed by intraoperative nerve monitoring. Histopathology confirmed the diagnosis of vagal schwannoma. This case highlights the importance of a multidisciplinary approach, thorough preoperative imaging, and meticulous intraoperative technique in achieving complete resection with minimal morbidity.

## Introduction

Schwannomas are slow-growing, encapsulated nerve sheath tumors originating from Schwann cells that account for 5% of benign soft tissue tumors [1]. These tumors arise from any myelinated peripheral, autonomic, or cranial nerve except the optic and olfactory nerves, with peak incidence between 20 and 50 years [2]. Vagus nerve schwannomas, typically located in the parapharyngeal space, represent 2%–5% of all nerve tumors [3, 4]. Clinically, cervical vagal schwannomas are mainly asymptomatic (68.2%). However, hoarseness due to vocal cord palsy is often seen in the later stages. Additionally, paroxysmal coughing during palpation along the medial sternocleidomastoid (SCM) border is pathognomonic for vagal schwannoma [5, 6]. Magnetic resonance imaging (MRI) is the most sensitive and specific investigation for diagnosis and preoperative assessment [7]. Complete surgical resection remains the primary treatment, although management is challenging due to nonspecific presentation and risk of intraoperative vagal nerve injury causing postoperative deficits [8].

Herein, we present a case of a 28-year-old Asian male with cervical vagal schwannoma who underwent nerve-sparing resection with complete functional recovery.

## Case presentation

A 28-year-old Asian male presented to the General Surgery clinic with a fast-growing right-sided neck swelling of 1 week’s duration. A 3 cm hard mixed mass was palpated. He was clinically stable with an unremarkable systemic examination. The patient was referred for diagnostic ultrasonography (US).

Ultrasound of the thyroid and neck revealed a well-defined heterogeneously hyperechoic lesion measuring 2.4 × 1.3 × 2.8 cm at level III of the right neck, without significant Doppler vascularity. A branchial cleft cyst was initially suspected, and MRI was performed for further evaluation. MRI demonstrated a well-defined, markedly enhancing lesion in the right carotid space measuring 30 × 24 × 20 mm (craniocaudal × transverse × anteroposterior). The lesion was hypointense on T1-weighted images, markedly hyperintense on T2-weighted images, showed diffusion restriction, avid post-contrast enhancement with multiple non-enhancing foci, and internal flow voids. The right common carotid artery was displaced anteriorly, and the imaging differential included glomus vagale and vagal schwannoma. The patient was referred to neurosurgery. Computed tomography (CT) angiography demonstrated a 3.0 × 2.5 × 2.0 cm enhancing right carotid space mass at C4–C5, displacing the common carotid artery anteriorly and internal jugular vein anterolaterally. The lesion contacted the transverse processes of C4 and C5 without extending to the carotid bifurcation, and there was no vascular invasion. After discussing treatment options, the patient consented to surgery (Fig. 1).

**Figure 1:**
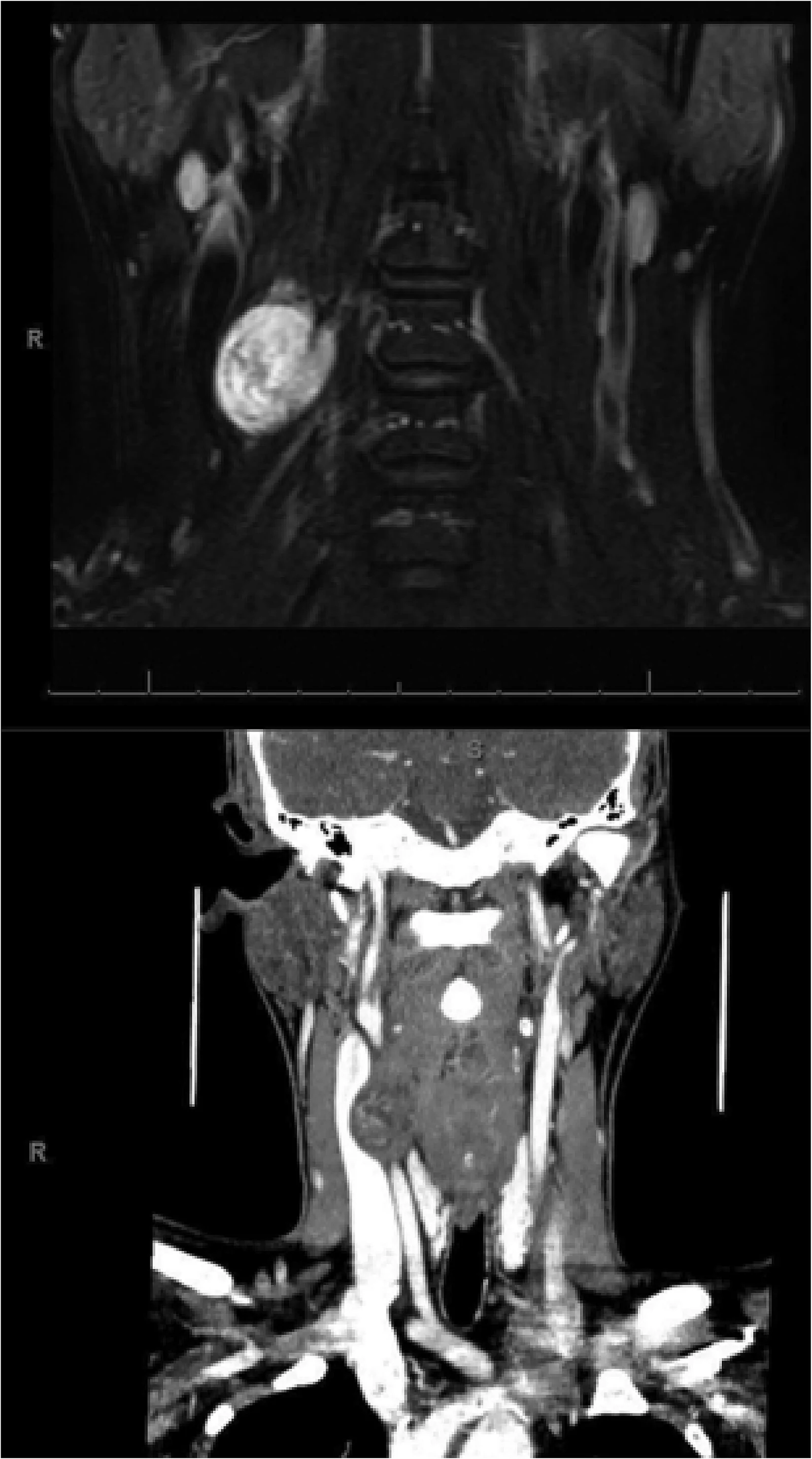
Preoperative MRI and CT angiography of the right cervical vagal schwannoma.

Intraoperative approach included patient lying in the supine position with a slight neck tilt and extension, facing the contralateral side. Surface anatomy skin markings were drawn outlining the mass for excision and the borders of the SCM as reference points (Fig. 2). A lateral transverse approach was agreed upon jointly by neurosurgery and vascular surgery, and vascular preparedness (standby carotid shunt, Dacron patch graft, and Prolene sutures) was arranged given the close relationship of the mass to the carotid sheath. Following infiltration with 1% lidocaine, a 5 cm transverse incision was made lateral to the SCM, and dissection proceeded through the platysma. Medial retraction of the SCM exposed the carotid sheath, which was carefully reflected to avoid vagal stimulation (Fig. 3). The internal jugular vein was preserved, and the common carotid artery was identified displaced laterally and inferiorly. The tumor was attached to the medial wall of the cervical vertebra at C4 and proximally to the vagus nerve. Using blunt and sharp dissection, it was excised en bloc with its capsule (Figs 4 and 5). Continuous intraoperative nerve monitoring demonstrated no irritation of the facial nerve, vagus nerve, or vocal cords. Estimated blood loss was 10 ml, with no transfusion or intraoperative complications. Closure was performed in layers using 2–0 Vicryl for the platysma, 3–0 Vicryl for soft tissues, and 3–0 Monocryl with Steri-Strips for skin closure. Histopathology confirmed vagal schwannoma. Postoperatively, serial neurological examinations and swallow assessment were normal, with no hoarseness, dysphagia, or other vagal deficits. The patient recovered uneventfully and was discharged the following day.

**Figure 2:**
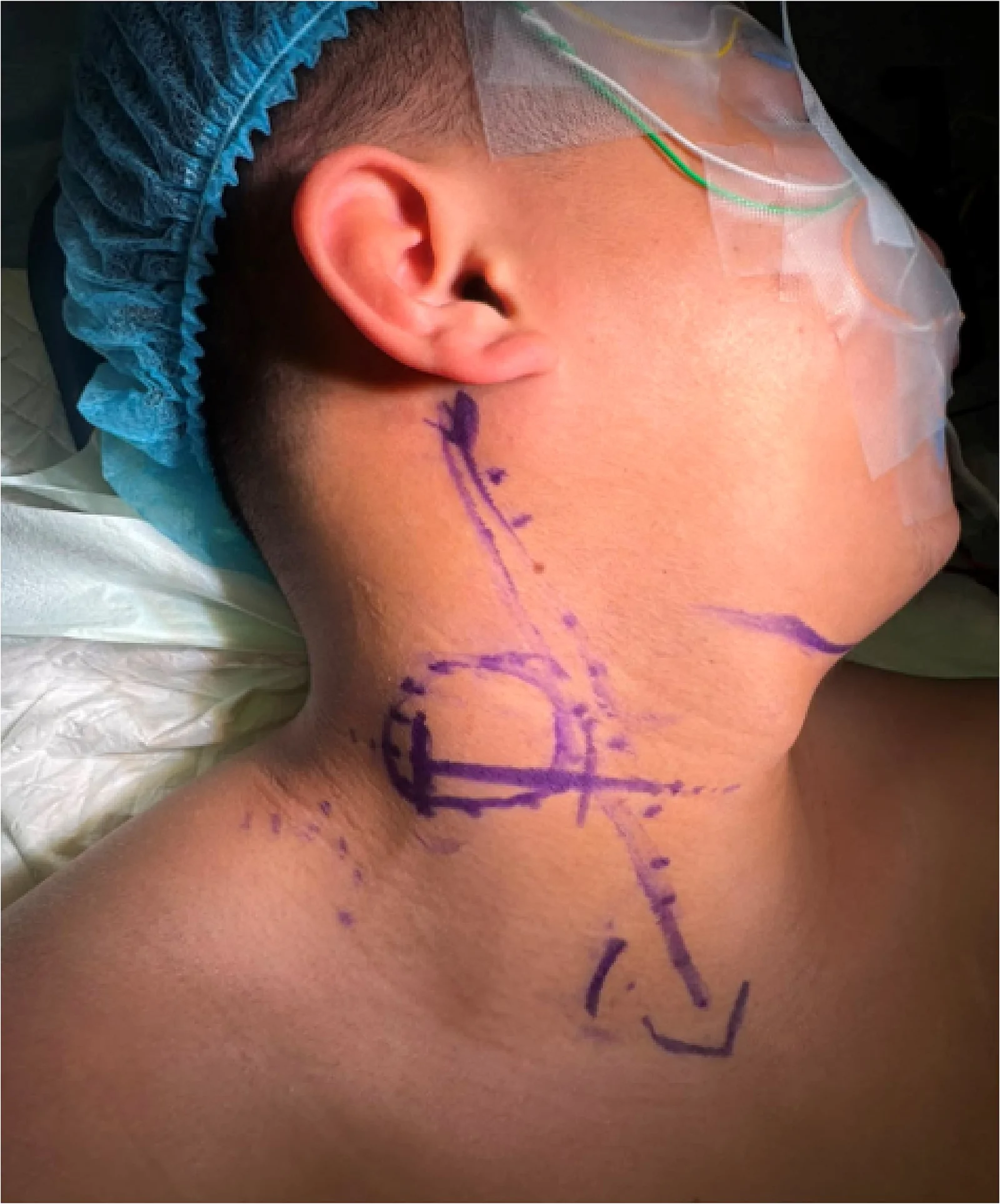
Preoperative surface anatomy markings delineating the tumor location and SCM borders for surgical planning.

**Figure 3:**
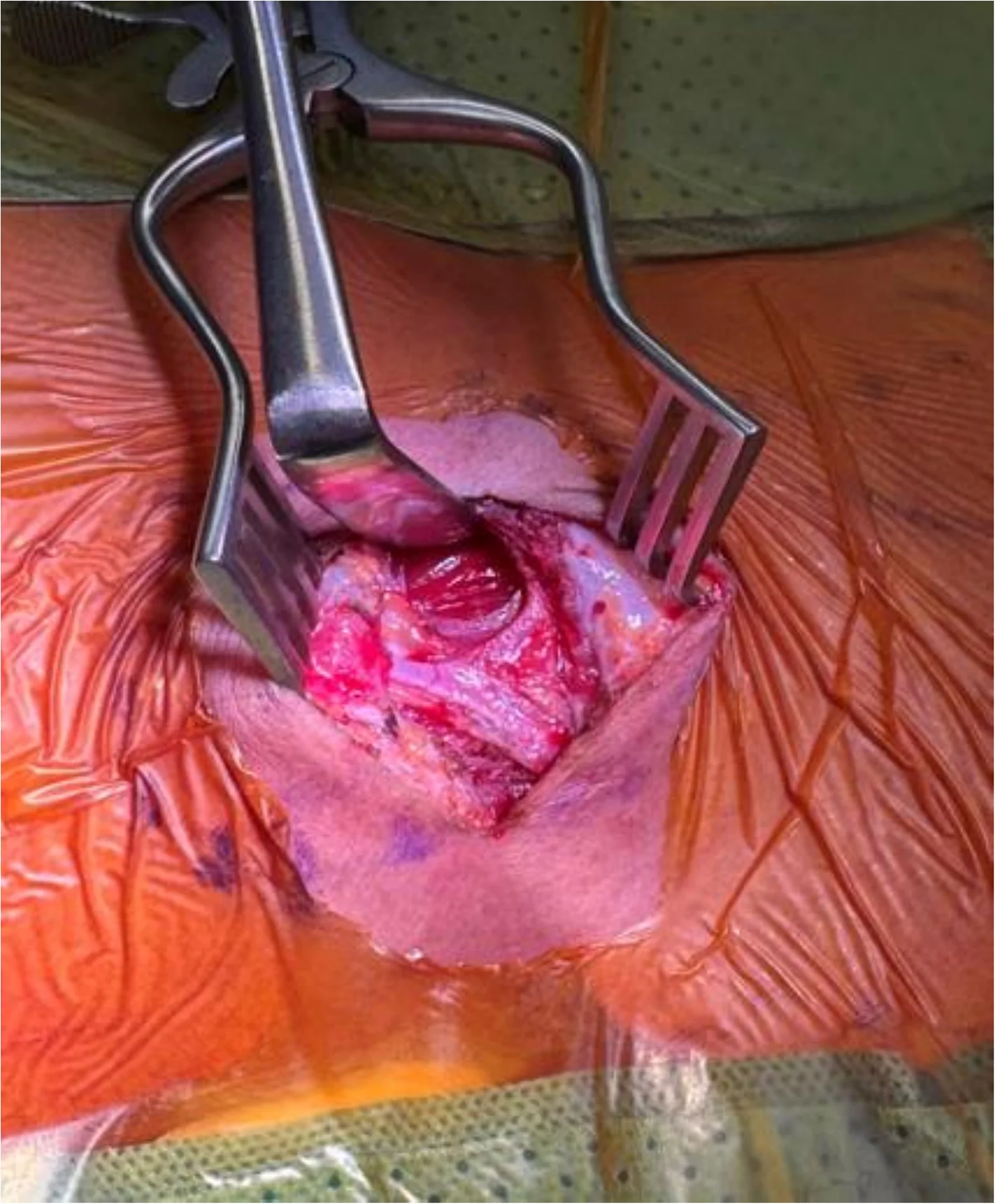
Intraoperative view demonstrating initial surgical dissection through the platysma and exposure of the carotid sheath.

**Figure 4:**
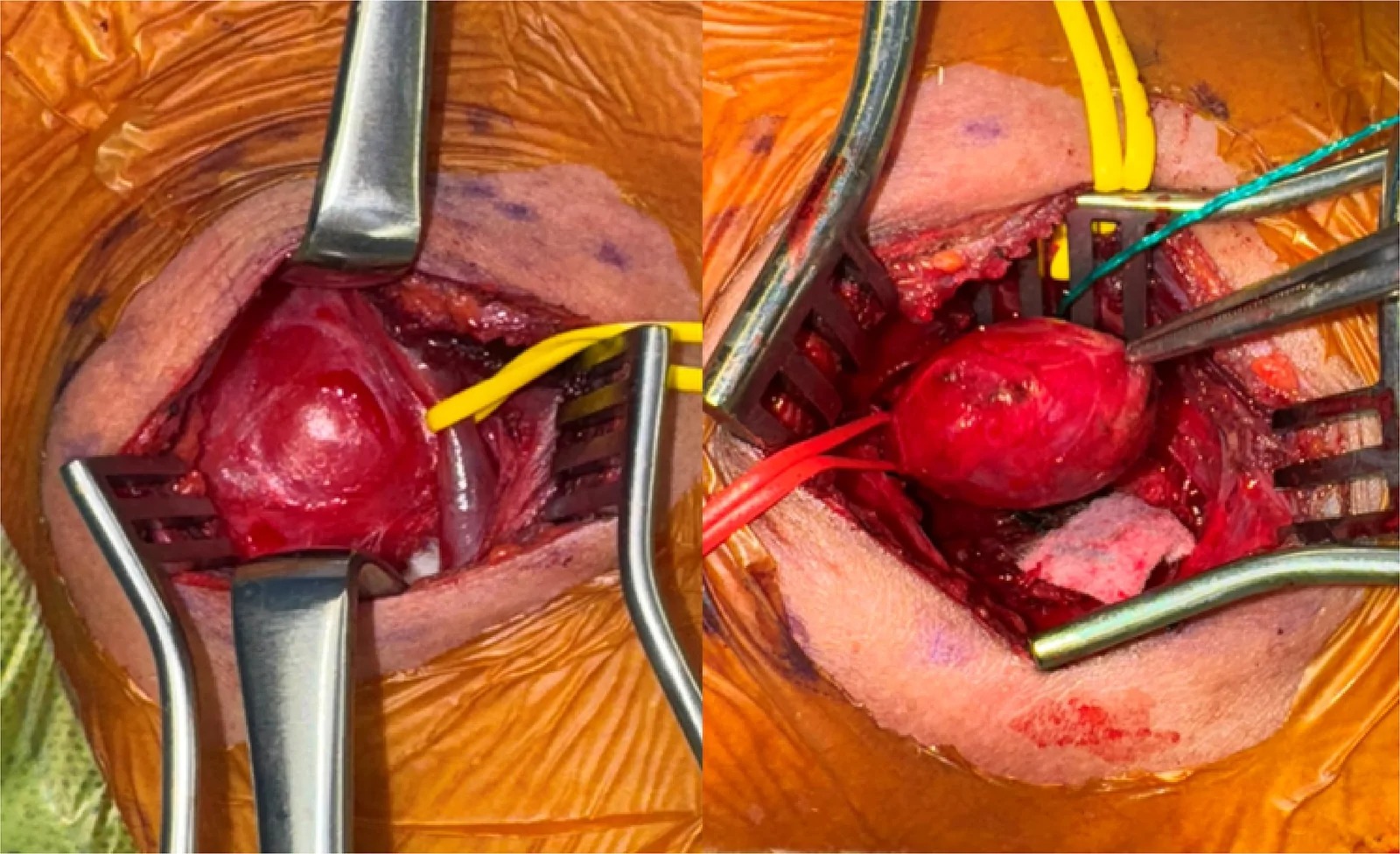
Intraoperative view showing the encapsulated vagal schwannoma before en bloc resection, with surrounding neurovascular structures protected by colored vessel loops (red: carotid artery; yellow and teal: adjacent neurovascular structures).

**Figure 5:**
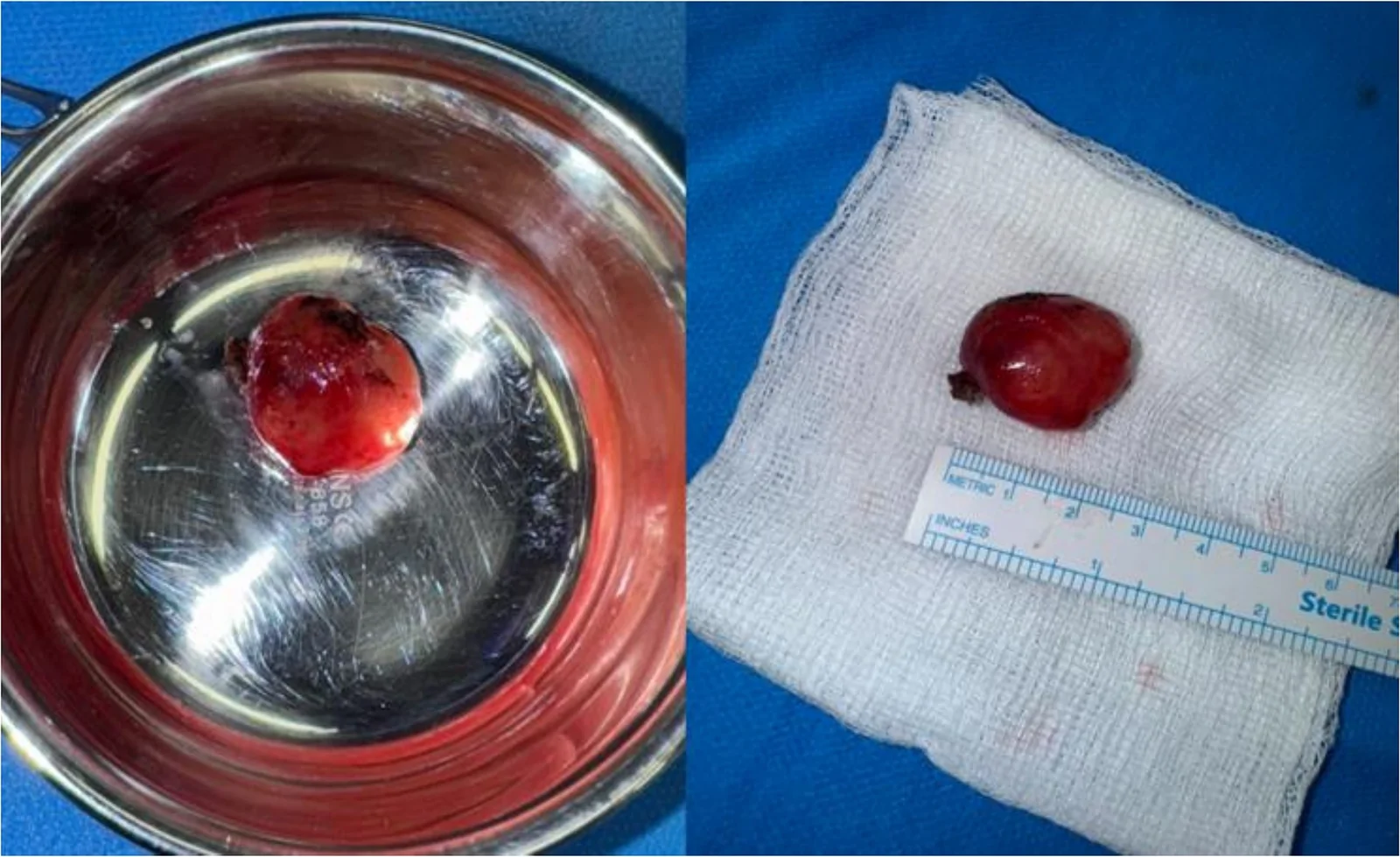
Gross specimen of the resected vagal schwannoma with a sterile metric ruler demonstrating tumor dimensions of ~2.4 cm.

## Discussion

Schwannomas pose a diagnostic challenge due to nonspecific clinical presentation. A unilateral asymptomatic neck mass may be misinterpreted as lymphadenopathy, carotid body tumor, branchial cyst, thyroid lesions, or parotid lesions [6, 7]. US and fine-needle aspiration often fail to distinguish neurogenic tumors and provide poor diagnostic yield [8]. Open biopsies risk vagal stimulation, with reported cases of severe bradycardia and cardiac arrest [9]. Therefore, MRI is the investigation of choice for preoperative evaluation, typically showing a well-circumscribed tumor between the internal jugular vein and carotid artery [10].

Management includes conservative observation or surgical excision. Their slow growth supports watchful waiting with serial imaging [5]. This approach is reserved for asymptomatic, older, or surgically high-risk patients [11]. However, tumor growth rates are higher in younger (<50 years) patients, making prolonged observation less suitable [5].

The second management strategy is surgical excision. Complete surgical excision is the treatment of choice for vagal nerve schwannomas [12]. However, due to their anatomical location, resection is often associated with significant morbidity [11]. In a systematic review by Suárez *et al*., only 11 of 254 surgically treated patients demonstrated preserved vagal nerve function, with full preservation achieved in just three cases [13]. Similarly, Valentino *et al*. reported that complete excision required sacrifice of the involved nerve in 56% of cases, while among the remaining 44%, permanent deficits occurred in 64% and transient deficits in 29% [14, 15]. Careful intraoperative management using nerve stimulators and continuous neurophysiological monitoring can significantly reduce postoperative complications [7].

In this case, the patient was young, otherwise healthy, with an asymptomatic lesion. Preoperative imaging showed clear margins and limited local invasion, favoring complete resection and nerve preservation. Through multidisciplinary planning, the tumor was completely excised with preserved vagal function.

Although complete excision remains the standard of care, proximity to critical neurovascular structures poses significant risks of postoperative functional impairment, including hoarseness, vocal cord paralysis, and other vagal deficits. Patient age, comorbidities, tumor characteristics, and symptom burden must be considered when choosing between observation and surgery. Thorough counseling is essential to balance symptom control, quality of life, and surgical morbidity.
